# FASN multi-omic characterization reveals metabolic heterogeneity in pancreatic and prostate adenocarcinoma

**DOI:** 10.1186/s12967-023-03874-5

**Published:** 2023-01-17

**Authors:** Ugo Chianese, Chiara Papulino, Ahmad Ali, Fortunato Ciardiello, Salvatore Cappabianca, Lucia Altucci, Vincenzo Carafa, Rosaria Benedetti

**Affiliations:** 1 Department of Precision Medicine, University of Campania “Luigi Vanvitelli”, L. De Crecchio 7, 80138 Naples, Italy; 2 Biogem Institute of Molecular and Genetic Biology, 83031 Ariano Irpino, Italy; 3 IEOS, Institute for Endocrinology and Oncology “Gaetano Salvatore”, 80131 Naples, Italy

**Keywords:** FASN, Pancreatic adenocarcinoma, Prostate adenocarcinoma, Metabolism, Proliferation

## Abstract

**Background:**

Pancreatic ductal adenocarcinoma (PDAC) and prostate cancer (PCa) are among the most prevalent malignant tumors worldwide. There is now a comprehensive understanding of metabolic reprogramming as a hallmark of cancer. Fatty acid synthase (FASN) is a key regulator of the lipid metabolic network, providing energy to favor tumor proliferation and development. Whereas the biological role of FASN is known, its response and sensitivity to inhibition have not yet been fully established in these two cancer settings.

**Methods:**

To evaluate the association between FASN expression, methylation, prognosis, and mutational profile in PDAC and PCa, we interrogated public databases and surveyed online platforms using TCGA data. The STRING database was used to investigate FASN interactors, and the Gene Set Enrichment Analysis platform Reactome database was used to perform an enrichment analysis using data from RNA sequencing public databases of PDAC and PCa. In vitro models using PDAC and PCa cell lines were used to corroborate the expression of FASN, as shown by Western blot, and the effects of FASN inhibition on cell proliferation/cell cycle progression and mitochondrial respiration were investigated with MTT, colony formation assay, cell cycle analysis and MitoStress Test.

**Results:**

The expression of FASN was not modulated in PDAC compared to normal pancreatic tissues, while it was overexpressed in PCa, which also displayed a different level of promoter methylation. Based on tumor grade, FASN expression decreased in advanced stages of PDAC, but increased in PCa. A low incidence of FASN mutations was found for both tumors. FASN was overexpressed in PCa, despite not reaching statistical significance, and was associated with a worse prognosis than in PDAC. The biological role of FASN interactors correlated with lipid metabolism, and GSEA indicated that lipid-mediated mitochondrial respiration was enriched in PCa. Following validation of FASN overexpression in PCa compared to PDAC in vitro, we tested TVB-2640 as a FASN inhibitor. PCa proliferation arrest was modulated by FASN inhibition in a dose- and time-dependent manner, whereas PDAC proliferation was not altered. In line with this finding, mitochondrial respiration was found to be more affected in PCa than in PDAC. FASN inhibition interfered with metabolic signaling causing lipid accumulation and affecting cell viability with an impact on the replicative processes.

**Conclusions:**

FASN exhibited differential expression patterns in PDAC and PCa, suggesting a different evolution during cancer progression. This was corroborated by the fact that both tumors responded differently to FASN inhibition in terms of proliferative potential and mitochondrial respiration, indicating that its use should reflect context specificity.

**Supplementary Information:**

The online version contains supplementary material available at 10.1186/s12967-023-03874-5.

## Background

Cancer is characterized by metabolic reprogramming, and different cancer types can display very different metabolic phenotypes based on metabolic plasticity, tissue origin, and tumor microenvironment, highlighting the existence of tumor-specific bioenergetic circuits [1–4]. Warburg first described the metabolic behavior of some cancers that showed increased lactic fermentation compared to traditional mitochondrial respiration, thus allowing ATP production and biomass required for tumor growth [5, 6] Altered metabolic processes contribute to the development of cancer, which, as a system with a high rate of proliferation, needs a large amount of fuel to maintain the activity of its biological processes [7, 8]. ATP, the energy currency and metabolic byproduct, is required for a variety of cellular processes, including DNA replication and the proper function of the cytoskeletal system [9, 10]. Pancreatic ductal adenocarcinoma (PDAC) and prostate cancer (PCa) are among the leading causes of cancer deaths worldwide [11–13]. In 2020, there were approximately 1.41 million new PCa cases at global level, according to GLOBOCAN [14] while PDAC is one of the cancer types with the highest lethality and is estimated to become the second leading cause of cancer-related deaths by 2030 [15–17]. Interestingly, evidence of altered energetic pathways is reported for these two tumors. In PDAC, KRAS oncogenic signaling and inactivation of tumor suppressors are directly related to altered glycolysis, recognized as the main metabolic alteration in pancreatic cancer [18–21]. In contrast, prostate tumors seem to favor increased oxidative phosphorylation, and fatty acid (FA) production appears to be linked to prostate carcinogenesis [22–24]. In terms of proliferation and survival, de novo FA biogenesis provides tumors with a competitive advantage [25]. FA synthase (FASN), a crucial enzyme in lipogenesis, is responsible for catalyzing the synthesis of the long-chain saturated FA palmitate, used as source for ATP production [26, 27]. In recent years, studies investigating antitumor strategies targeting metabolism regulation have increased, and FASN inhibition has shown promise in targeted cancer therapy [28–30]. However, little is known about cancer cell sensitivity to FASN inhibitors, thus creating a bottleneck for their therapeutic application. Antimetabolic strategies are currently under investigation in PDAC and PCa, and clinical trials are evaluating how the disruption of lipid signaling may contribute to an improvement in patient outcomes [24, 31].

Here, we investigated differences in FASN expression in PDAC and PCa using a multi-omic approach. We also explored the effect of FASN inhibition on proliferation and mitochondrial respiration in these two cancer settings. Our results show a different role for FASN in the two tumor types, suggesting a different bioenergetic evolution. FASN expression differs significantly in PDAC and PCa, with a worse prognosis associated with PCa and high expression. FASN protein–protein interaction analysis indicated that its interactors are mainly involved in mitochondrial respiration. In line with this finding, Gene Set Enrichment Analysis (GSEA) on RNA sequencing (RNA-seq) TCGA data identified mitochondrial respiration- and lipid-related genes as differentially expressed and particularly enriched in PCa. This was corroborated by the fact that the two tumors responded differently to FASN inhibition in terms of proliferative potential and mitochondrial respiration, suggesting that FASN inhibition might represent a more effective therapeutic strategy in PCa.

## Methods

### Exploration of gene expression patterns in different cancers

Gene Expression across Normal and Tumor tissue (GENT) is a web-accessible resource where gene expression patterns in different human cancers and normal tissues are explored [32]. To obtain differential expression patterns of FASN in normal vs cancer tissues, we applied a set of default parameters on datasets, samples, and probes.

### Extensive analysis of methylation expression data

UALCAN is a publicly available tool for the in-depth analysis of TCGA gene expression data [33]. Using the UALCAN database, we analyzed the methylation profile of the FASN promoter in PDAC, PCa, and normal tissues.

### Chemicals

TVB-2640 was purchased from Selleckchem Chemicals (Houston, TX, USA, #S9714) and was used at a final concentration of 1 μM, 5 μM, 10 μM, 25 μM, and 50 μM for proliferative assays, and 50 μM for the Mito Stress Test.

### Cell culture

PL-45, SW1990, LNCaP, and C4-2 cell lines were purchased from ATCC (Milan, Italy). PL-45 and SW1990 were grown in Dulbecco’s Modified Eagle’s Medium (DMEM; Euroclone, Milan, Italy, #ECB7501L), supplemented with 10% heat-inactivated fetal bovine serum (FBS; Sigma-Aldrich, St. Louis, USA, #F7524), antimicrobials (100 U/mL penicillin, 100 µg/mL streptomycin [Euroclone, ECB3001D], 250 ng/mL amphotericin B [Euroclone, ECM0009D], and 2 mM l-glutamine [Euroclone, ECB3000D]). LNCaP and C4-2 cells were grown in Roswell Park Memorial Institute culture medium (RPMI; Euroclone, ECB9006L), supplemented with 10% heat-inactivated FBS (Sigma-Aldrich, F7524), antimicrobials (100 U/mL penicillin, 100 µg/mL streptomycin [Euroclone, ECB3001D], 250 ng/mL amphotericin B [Euroclone, ECM0009D], and 2 mM l-glutamine [EuroClone, ECB3000D]), and 1% essential amino acids solution (MEM; Euroclone, ECB3054D). All cell lines were cultivated at 37 °C with 5% CO_2_ and were checked for mycoplasma contamination using EZ-PCR Mycoplasma Test Kit (Biological Industries, Connecticut, USA; #20-700-20).

### Western blot analysis

Cell pellets were suspended in lysis buffer (50 mmol/L Tris–HCl pH 7.4, 150 mmol/L NaCl, 1% NP40, 10 mmol/L NaF, 1 mmol/L PMSF, and protease inhibitor cocktail). Next, the lysis reaction was carried out for 15 min at 4 °C, samples were centrifuged at 13,000 rpm for 30 min at 4 °C, and protein concentration quantified by Bradford assay (Bio-Rad Protein Assay Dye Reagent Concentrate, Bio-Rad, Hercules, CA, USA, #5000006). A total of 30 μg of each sample was loaded on 8% polyacrylamide gels and electro-blotted on nitrocellulose membranes. Immunoreactive signals were detected with the horseradish peroxidase-conjugated secondary antibody (Bio-Rad, #1705046, #1705046). Primary antibodies were: FASN (C20G5), GAPDH (D16H11), ACSL1 (D2H5), Lipin 1 (D2W9G), purchased from Cell Signaling Technology (Danvers, MA, USA). Tubulin (sc-5286) was purchased from Santa Cruz Biotechnology. All antibodies were used according to the manufacturer’s instructions. Semi-quantitative analysis was performed using ImageJ software (version 1.44), and the relative abundance is reported in Fig. 4A.

### Cell viability assay

Cell viability was determined on PL-45, SW1990, LNCaP, and C4-2 cell lines using thiazolyl blue tetrazolium bromide [3-(4,5-dimethylthiazol-2-yl)-2,5-diphenyltetrazolium bromide] (MTT; Sigma-Aldrich, Schnellendorf, Germany) assay, following the manufacturer’s instructions. A total of 8 × 10^3^ cells/well were plated in a 96-well plate and then treated with TVB-2640; experiments were performed in triplicates and repeated for three times. The FASN inhibitor TVB-2640 was used at different concentrations (1 μM, 5 μM, 10 μM, 25 μM, 50 μM) for 24 h, 48 h, and 72 h. Absorbance was read at a wavelength of 570 nm with a TECAN M200 reader (Tecan, Männedorf, Switzerland).

### Cellular mitochondrial stress

Metabolic status was investigated on a Seahorse XF96 Analyzer (Agilent Technologies, Santa Clara, CA, USA) with standard 96-well Seahorse microplates. A Mito Stress Test Kit (Agilent Technologies, #103015) was used to assess oxygen consumption ratio (OCR) after TVB-2640 treatment. In brief, 8 × 10^3^ cells were seeded into plates 24 h prior to analysis. The medium was then replaced with 175 µl of non-buffered RPMI and DMEM containing 10 mM glucose, 2 mM glutamine, and 1 mM pyruvate. The cells were then treated with 50 μM TVB-2640 for 6 h at 37 °C and incubated in a CO_2_-free incubator at 37 °C for 1 h to allow for temperature and pH equilibration before being loaded into the XF96 Analyzer. The injection sequence was programmed as follows: 1st, oligomycin (1 µM at final concentration); 2nd, carbonyl cyanide m-chlorophenylhydrazone (FCCP; 1 µM at final concentration); 3rd, rotenone and antimycin A (1 µM and 0.5 µM at final concentrations, respectively). Data were analyzed with Wave software (version 2.2.0, Seahorse Bioscience, Agilent Technologies, Santa Clara, CA, USA). Experiments were performed in triplicates. p-values were calculated using t-test. Statistical significance is expressed as *p < 0.05. Standard deviations are reported as error bars.

### Analysis of protein–protein interaction and functional protein partners

STRING was used for protein–protein interaction analysis. STRING is an extensive database of functional association data documenting both physical and functional protein–protein interactions [34]. This database was used to determine the functional protein partners of FASN.

### Gene set enrichment analysis

Enrichment analysis was performed with GSEA (version 4.3.1) using Reactome as the gene set database. PDAC and PCa RNA-seq from the TCGA database were uploaded as normalized counts.

### Identification of mutations

The cBioPortal for Cancer Genomics is a web platform for exploring and analyzing cancer genomics datasets [35, 36]. In this study cBioPortal was used to explore mutations of FASN with defined parameter settings.

### FASN-SREBF1 correlation

Expression values of FASN and SREBF1, as positive regulator of FASN transcription, reported as TPM were downloaded from UALCAN using the TCGA database. Pearson correlation was evaluated with GraphPad Prism (version 8.3.0).

### Bulk RNA-seq analysis

PDAC and PCa TCGA RNA-seq data were downloaded from UCSC Xena. Statistical analyses were performed with DESeq2 [37] using R (version 3.6.4). To compare sample subgroups in terms of gene expression, the DESeq2 package that utilizes a negative binomial model was used to detect differentially expressed genes from count data. Expression differences in genes were considered statistically significant if the p-value was < 0.05.

### Kaplan–Meier analyses analysis

UALCAN and cBioportal platform have been used for survival analysis of PDAC and PCa patients based on FASN expression. High expression group collects samples with gene expression values equal to or more than the 3rd quartile value and Low/Medium expression group, samples with gene expression values less than the 3rd quartile.

### Colony formation assay

LNCaP were treated with TVB-2640 50 μM for 72 h. Then, the cells medium was changed and cells were cultured for additional 7 days in a drug-free medium. Colonies were stained with crystal violet (abs 595 nm) and counted to assesses the proliferative capability.

### Quantification of lipids with oil red o Staining

LNCaP were plated in 12-well and treated with TVB-2640 at 50 μM for 24 h. Cells were fixed with 4% formalin in PBS for 1 h and rinsed with distilled water at RT. The fixed cells were treated with 60% isopropyl alcohol for 5 min and then stained with 0.3% Oil Red O in 60% isopropyl alcohol solution for 15 min at RT and subsequently washed with distilled water. Lipis droplets were quantified by isopropanol extraction for 15 min and absorbance measured with Infinite M1000 microplate reader (TECAN) at 540 nm.

### Cell cycle and cell death analysis

Treated and untreated LNCaP cells were (2 × 10^5^ cells/mL) harvested with PBS, centrifuged at 1200 rpm for 5 min, and resuspended in 500 μL of a hypotonic solution (1X PBS, 0.1% sodium citrate, 0.1% NP-40, RNAase A, and 50 mg/mL PI). Cell death was studied by evaluating hypodiploid sub-G1 peak on fixed cells and PI incorporation on live cells to assess DNA fragmentation (early apoptotic event) and dead-cell membrane permeabilization (late apoptotic event), respectively. For sub-G1 evaluation, samples were prepared as described above. For PI evaluation, cells were plated (2 × 10^5^ cells/mL) and treated with TVB-2640 at 50 μM for 24 h. After treatment, cells were harvested with PBS, centrifuged at 1200 rpm for 5 min, and resuspended in 500 μL 1X PBS and 0.2 mg/mL PI. The results were acquired on a BD Accuri TM C6 flow cytometer system (BD Biosciences New Jersey, U.S.A.).

## Results

### FASN expression in PDAC and PCa compared to normal tissue

**Fig. 1:**
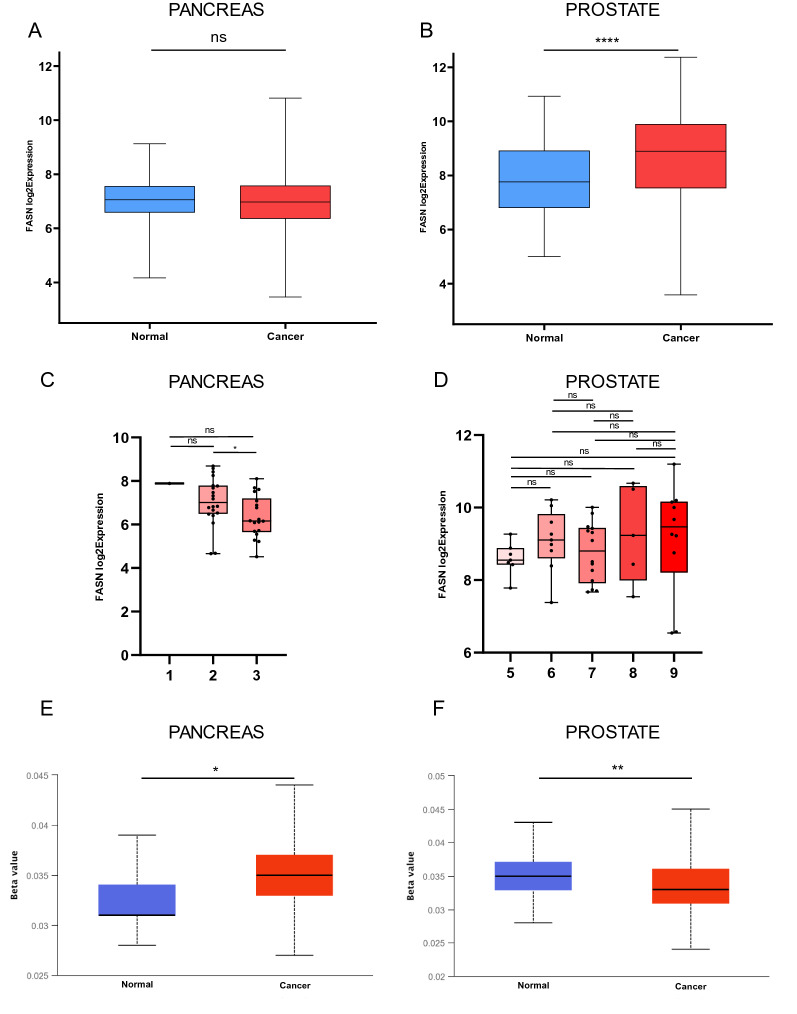
**A** Fatty acid synthase (FASN) transcription levels in normal pancreatic tissue and PDAC tissue from GLP96 and GLP570 databases. Statistically significant cal culated with paired t-test. **B** Statistically significant FASN transcription levels in normal prostate tissue and PCa tissue from GLP96 and GLP570 (**** < 0.0001) calculated with paired t-test. **C** FASN expression based on grade in PDAC samples. Statistical significance has been calculated as two-sample test across each tumor grade, (*p < 0.05) (ns not significance). **D** FASN expression based on grade in PCa tissue. Statistical significance has been calculated as two-sample test across each tumor grade (ns not significance). **E** Statistically significant FASN methylation levels in normal pancreatic tissue and PDAC tissue p < 0.05) calculated with paired t-test. **F** Statistically significant FASN methylation levels in normal prostate tissue and PCa tissue (**p < 0.01) calculated with paired t-test. Boxes indicate the median, and 25th and 75th percentiles. Red boxes indicate tumor tissues; blue boxes indicate normal tissues

FASN expression was investigated in normal and tumor tissues by analyzing microarray data from two different databases, GLP96 and GLP570 (Additional file 1: Figure S1). In total, 484 PDAC samples (normal = 119, cancer = 365) and 762 PCa samples (normal = 142, cancer = 620) were analyzed (Table 1) (Additional file 2: Table S1). FASN was differently regulated in PDAC and PCa compared to their normal tissue (NT) counterparts. Our analysis revealed no statistical difference in FASN expression in PDAC and NT (Fig. 1A), whereas FASN resulted statistically overexpressed in PCa compared to NT (Fig. 1B). A more detailed analysis based on tumor grade showed a significant reduction in FASN expression in advanced stage PDAC (Fig. 1C). An increase in expression of FASN was observed in PCa (Fig. 1D), although the result was not statistically significant. To correlate FASN expression with the methylation profile at its promoter in PDAC-NT and PCa-NT, we analyzed the methylation levels of FASN using the UALCAN platform with the TCGA database. Methylation levels were significantly higher in PDAC than in NT (Fig. 1E). In contrast, methylation of FASN was significantly downregulated in PCa compared to NT (Fig. 1F). Taken together, the reduction in FASN expression observed in PDAC was associated with increased methylation at its promoter, whereas the opposite was found in PCa, where higher expression levels of FASN were coupled with lower methylation. These findings suggest different roles and functions for FASN in PDAC and PCa systems.

**Table 1:** Microarray datasets used for FASN expression analysis of PDAC and PCa

Pancreas									
GSE1133	[38]	GSE16515	[39]	GSE2109	[40]	GSE9599	[41]	GSE43346	[42]
GSE11907	[43]	GSE17891	[44]	GSE22780	[40]	E-AFMX-5	[40]	GSE46385	[45]
GSE12630	[46]	GSE18670	[47]	GSE2361	[48]	GSE42952	[49]	GSE52171	[50]
GSE15471	[51]	GSE19281	[52]	GSE2719	[53]	GSE71989	[54]	GSE60601	[40]
GSE15932	[55]	GSE19650	[56]	GSE27890	[40]	GSE7307	[40]	GSE42252	[57]
GSE34111	[58]	GSE49515	[59]	GSE32688	[60]	GSE43288	[61]	GSE42404	[62]
GSE36076	[40]	GSE50570	[63]						
Prostate									
GSE1133	[38]	GSE30304	[64]	GSE25136	[65]	GSE7307	17088532	GSE2443	[66]
GSE12348	[67]	GSE30522	[68]	GSE26910	[69]	GSE8218	[70]	GSE6369	[40]
GSE12630	[46]	GSE3325	[64]	GSE2719	[53]	E-AFMX-5	[40]	GSE45016	[71]
GSE17951	[70]	GSE43346	[42]	GSE30174	[72]	E-MEXP-1327	[40]	GSE46602	[73]
GSE2109	GPL570	GSE2361	[48]	E-TABM-26	[40]

### FASN expression and mutational profile in PDAC and PCa

**Fig. 2:**
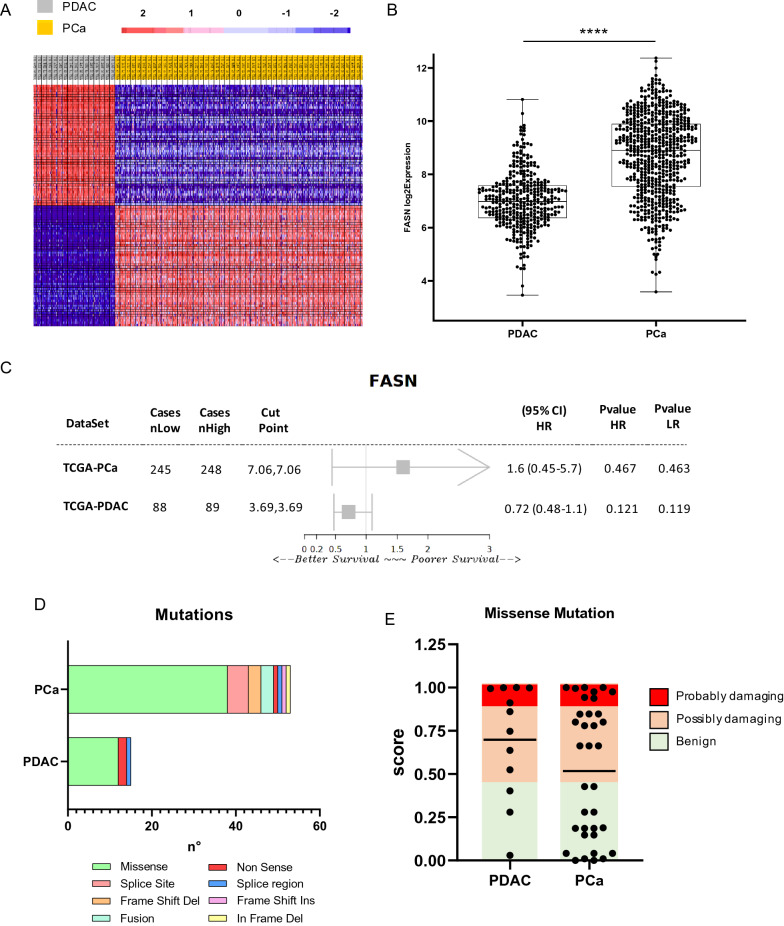
**A** Heatmap showing DEGs from an RNA-s eq data comparison between PDAC and PCa. **B** Box plot showing fold-change of FASN expression in PDAC and PCa FASN expression was derived from RNA-seq data (****p < 0.0001) calculated with paired t-test. **C** Forest plot showing survival index in PDAC and PCa for FASN from the web plat form Survival Genie. **D** FASNmutations in PDAC and PCa based on data obtained from cBioPortal. **E** Predictive score associated with protein damaging related to missensemutations PDAC and PCa based on data obtained from cBioPortal and Polyphen-2

To better investigate the difference in FASN expression in PDAC and PCa systems, we analyzed transcriptomic data using the TCGA database. In total, 182 PDAC and 551 PCa RNA-seq samples were analyzed and clustered into groups for DGE analysis (Fig. 2A). FASN was upregulated in PCa compared to PDAC (Fig. 2B) (Additional file 3: Table S2). These data were validated using several available platforms (Additional file 1: Figures S2 and S3), which all corroborated these differential expression patterns. The spliced transcripts of FASN also resulted overexpressed in PCa (Additional file 1: Figure S4). The prognosis in PDAC and PCa patients was then investigated based on its association with FASN expression levels. Although no significant correlation was observed between FASN expression and prognosis in PDAC and PCa (Additional file 1: Figure S5), different expression patterns were observed in the two cancer types. FASN expression was associated with a worse prognosis in PCa than in PDAC (Fig. 2C). The incidence of mutations in FASN was also low in both cancer types (Additional file 1: Figure S6). A total of 5 (990 samples) and 19 (9501 samples) studies investigating PDAC and PCa were analyzed, respectively; missense mutations were the most common mutations identified (Fig. 2D). In PDAC and PCa tumors, FASN single nucleus polymorphisms were associated with an altered functional property of 75% and 51.42%, respectively (Fig. 2E). Together, these data corroborate and strengthen the differential expression of FASN in PDAC and PCa, a higher expression of FASN was observed in PCa than in PDAC and correlated with a worse prognosis in this cancer setting (Additional file 4: Table S3).

### FASN protein interactions and their association with mitochondrial respiration and prognosis

**Fig. 3:**
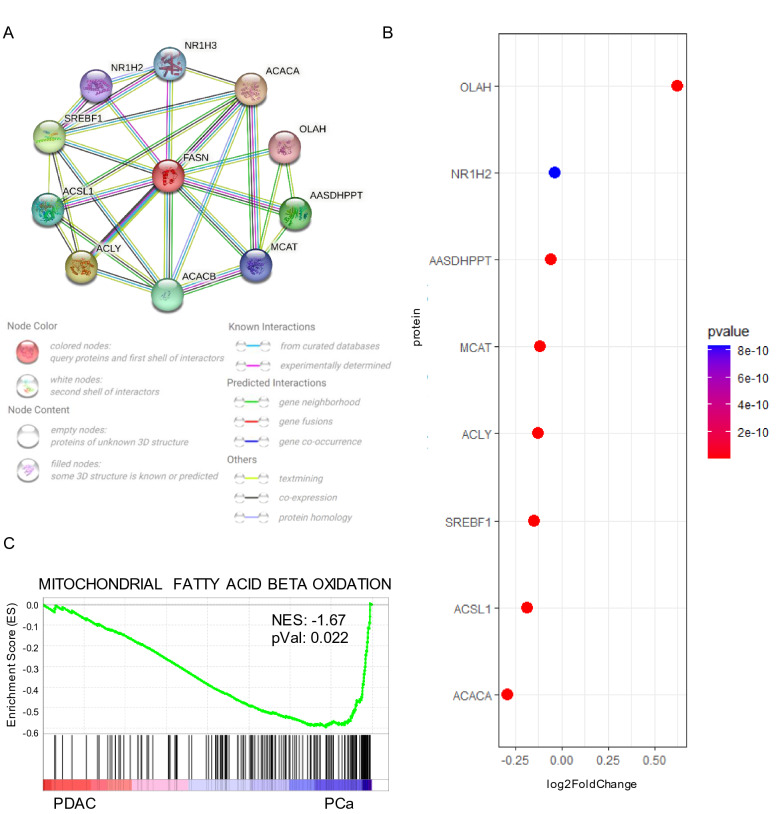
**A** predicted structural proteins essential for the functioning of FASN generated from STRING. Circles indicate nodes. Predicted functional interactors are shown as Top 10 based on score. **B** Expression of Top 10 FASN interactors in PDAC and Pca. **C** GSEA performed with Reactome gene set database matching pCA against PDAC

To clarify the difference in FASN expression in PDAC and PCa, we interrogated TRRUST2, a public database of transcription factors, and identified SREBF1 as a positive regulator of FASN transcription. FASN and SREBF1 expression data were analyzed from the UALCAN TCGA database (Additional file 5: Table S4) to correlate their co-expression. The analysis revealed a stronger correlation in PCa (Pearson correlation = 0.63) than in PDAC (Pearson correlation = 0.21) (Additional file 1: Figure S7). To better understand the biological context of FASN, its functional protein interactors were also investigated using STRING. The Top 10 list based on score (Additional file 6: Table S5) was mainly related to proteins involved in the metabolic processes (Fig. 3A), such as ACACA and SREBF1. Most of these interactors were overexpressed in PCa compared to PDAC (Fig. 3B), supporting the idea of a higher activity for the metabolic lipid branch in PCa. To validate this hypothesis, GSEA was performed using the Reactome database matching TCGA RNA-seq from PDAC and PCa data. Mitochondrial FA beta oxidation was statistically enriched in PCa (Fig. 3C). These findings support a functional role for FASN expression in PCa.

### FASN inhibition impacts on cell proliferation and mitochondrial respiration

**Fig. 4:**
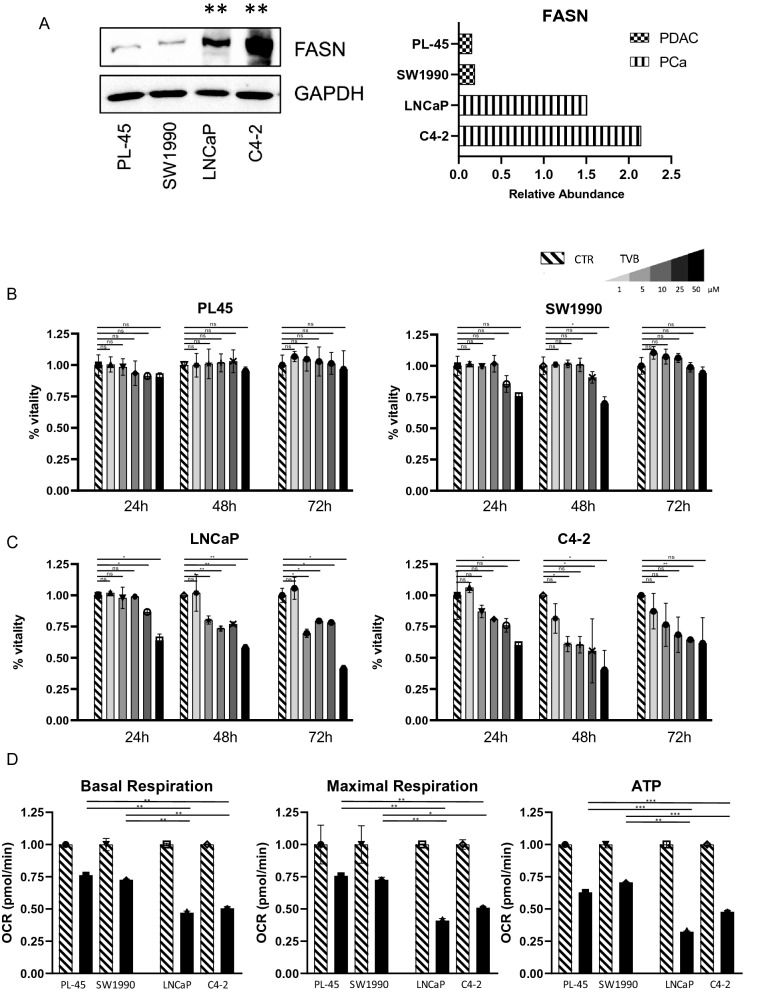
**A** FASN expression by Western blot analysis in PL-45, SW1990, LNCaP, and C4-2 cells with relative abundance. Statistical significance with two sample t-test between PDAC and PCa cell lines reported as **p < 0.01. **B** Viability assay in PDAC systems (PL-45 and SW1990). TVB-2640 was used at a final concentration of 1 μM, 5 μM, 10 μM, 25 μM, and 50 μM for 24 h, 48 h, and 72 h. **C** Proliferation assay in PCa systems (LNCaP and C4-2). TVB-2640 was used at a final concentration of 1 μM, 5 μM, 10 μM, 25 μM, and 50 μM for 24 h, 48 h, and 72 h. **D** Oxygen consumption rate (OCR) for basal respiration, maximal respiration, and ATP production in PL-45, SW1990, LNCaP, and C4-2 cells after TVB-2640 treatment at 50 μM for 6 h. Statistical significance with two sample t-test reported as *,**,*** p < 0.05, < 0.01, < 0.001

Having determined the differential FASN expression levels in PDAC and PCa, we performed a cell viability assay on four different cell lines, two for each tumor type. Interestingly, and in agreement with our data, Western blot analysis showed that FASN expression was lower in PL-45 and SW1990 PDAC cells, and much higher in LNCaP and C4-2 PCa cells (Fig. 4A). We also used a FASN inhibitor, TVB-2640, to investigate its potential effects on proliferation in PCa and PDAC systems. In line with the newly identified differential FASN expression levels, no significant activity was observed in PDAC cells (Fig. 4B), whereas a significant reduction in proliferation was observed in PCa cells (Fig. 4C). In agreement, the IC_50_ values for the TVB-2640-mediated effects showed that a higher concentration was required in PDAC cells to induce a proliferative arrest of 50% of the cell population compared to PCa cells (Additional file 1: Figure S8). Finally, to investigate the impact of FASN inhibition on mitochondrial respiration in the two cancer systems, we performed a Mito Stress Test. Intriguingly, the results indicated that FASN inhibition had a greater effect in PCa, where it was associated with a reduction in basal and maximal respiration and a drop in ATP production (Fig. 4D). Taken together, these data show that FASN is overexpressed in PCa and that its inhibition reduces proliferation by regulating mitochondrial respiration in PCa.

### FASN inhibition alters lipid signaling interfering with proliferative capability

**Fig. 5:**
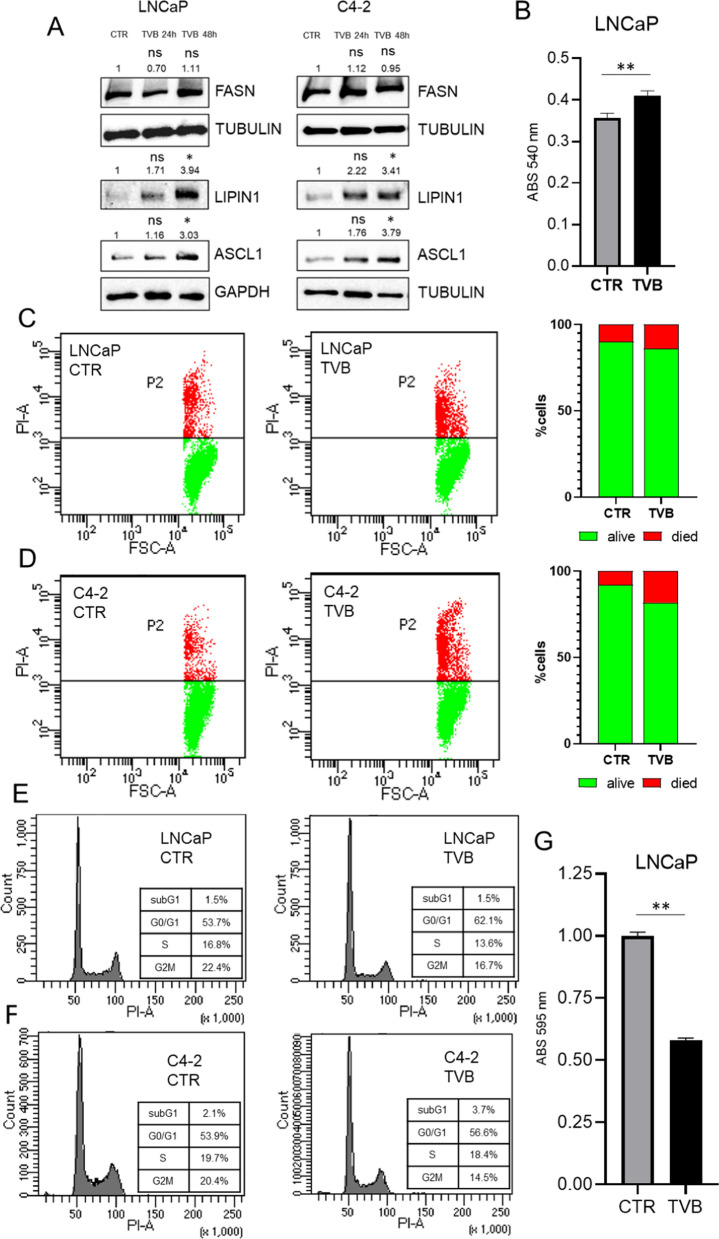
**A** Western blot analysis of FASN expression in LNCaP and C4-2 cells before and after treatment with TVB-2640 50 μM for 24 h and 48 h. Data were normalized based on control relative abundance. Statistical significance has been calculated with two saples t-test (treated and control) and reported as p** < 0.01. **B** Histograms reporting absorbance values of red oil assay in LNCaP cells before and after treatment with TVB-2640 50 μM for 72 h. Statistical significance calculated with two saples t-test and reported as p** < 0.01. **C**–**D** Effects of TVB-2640-related on propidium iodide (PI) incorporation, LNCaP and C4-2 cells were treated with TVB-2640 at 50 μM for 24 h. Graphs of PI distribution show the alive cells (green) and death cells (red). **E**–**F** Effects of TVB-2640-related on cell cycle regulation. LNCaP and C4-2 were treated with TVB-2640 at 50 μM for 24 h. Graphs show subG1, G0/G1, S and G2M phases. G Histograms reporting absorbance values of colony formation assay on LNCaP cells before and after treatment with TVB-2640 50 μM for 72 h. Statistical significance has been calculated with two saples t-test and reported as p** < 0.01

FASN inhibition has an impact on lipid metabolism, cell viability and cell cycle. Although TVB in a in- dependent manner did not alter FASN expression level, it was able to modulate two important lypogenesis-related proteins such as LIPIN1 and ASCL1, both FASN interactors (Fig. 5A). This effect was correlated to increased lipid accumulation (Fig. 5B) (Additional file 1: Figure S9) and with cellular proliferation and cell death (Fig. 5C and D). Cell cycle analysis indicated that FASN inhibition reduced the transition into S/G2M phases, and induced cell cycle arrest accumulating in G0/G1 phase as showed in Fig. 5E–F. These effects on cell cycle were also coupled with reduced colony formation capability (Additional file 1: Figures S10 and S5G). Taken together, these data show that FASN inhibition altered metabolic signaling pushing for lipid accumulation and affecting cell viability with an impact on the replicative processes.

## Discussion

The crosstalk between metabolic deregulation and cancer is one of the new frontiers for the identification of cancer biomarkers and the design of more effective therapeutic strategies [74, 75]. Some studies suggest a tumor-specific variability in energy that benefits progression of the disease, while intervention on the metabolic axis has been associated with an improvement in sensitivity to therapies [76] and relapse-free survival rates. Glutaminase inhibitors have shown efficacy in preclinical cancer models for triple-negative breast cancer [77], acute myeloid leukemia [78], and renal cell carcinoma [79]. The control of lipid homeostasis through regulation of circulating lipoprotein levels and cholesterol metabolism disruption have already reached clinical trials evaluating how lipid intake reduction impacts on disease progression in PCa. Indeed, as regulators of the LDL/HDL ratio, statins are under investigation in PCa (NCT02534376, NCT01821404, NCT04776889, NCT01992042, NCT00572468). Although less is known about the metabolic profile of PDAC, a clinical study currently in the recruiting stage aims to evaluate its impact (NCT04862260). A potentially new approach for lipid metabolic control is directed not at reducing the absorption (or balance) of circulating lipoproteins but at inhibiting FASN. TVB-2640 is the first FASN inhibitor to be investigated in clinical trials: nine studies are focusing on its potential use in monotherapy and/or combination therapy (NCT03808558, NCT02223247, NCT04352361, NCT03938246, NCT02980029, NCT04906421, NCT03032490411, NCT0317917517). Although these studies are still in the recruiting phase, preliminary results indicate a good tolerability and an improvement in anticancer action when used in combination against non-small cell lung cancer (NCT03808558) and breast cancer (NCT03179904). Despite these encouraging preliminary results, there is no single metabolic phenotypic classification in cancers, and further studies are needed to characterize the energy networks associated with tumor progression. Our data, obtained by multi-omic analyses, suggest that FASN might have a greater role as an oncogene and metabolic regulator in PCa than in PDAC. The differences we observed in FASN expression, promoter methylation, and sensitivity to direct inhibition in PCa and PDAC strongly suggest that a comprehensive multi-omic and integrated molecular analysis is necessary to select the the most appropriate cancer contexts to study at molecular and pathogenetic level. The, at least partial, link between differentially altered FASN expression and its promoter methylation also suggests an underlying crosstalk between genome and epigenome in the deregulation of FASN in cancer, which often seems unrelated to mutational processes. Although altered FASN expressions levels were not significantly associated with poor prognosis, the trend suggested that its overexpression might be. FASN overexpression in PCa might also lead to a stronger deregulation in lipid metabolism in PCa. Analyses carried out to identify the role of FASN in functional protein association networks also identified proteins involved in lipid metabolic activity; GSEA showed that lipid-mediated mitochondrial respiration was enriched in PCa, suggesting a role for FASN in metabolic deregulation occurring during PCa progression. Our in vitro results also show the potential anticancer action of FASN inhibition, which exhibited a strong antiproliferative effect in PCa, supporting the hypothesis that this different sensitivity might also be related to the onco-metabolic role of FASN in PCa. Our findings indicate that FASN inhibition was able to block cell proliferation in G0/G1 phase, increasing cell death and reducing colony formation capability in PCa. Furthermore, investigation on mitochondrial respiration also indicate that FASN inhibition leads to a marked reduction in energy production in PCa, altering metabolic signaling and causing lipid accumulation. According to other scientific evidences, it is reasonable to think that FASN inhibition alters the metabolic axis with lipid accumulation causing a condition known as lipotoxicity [80, 81] with a concurrent impairment of the replication machinery that block cell proliferation in G0/G1 phase, which has been previously described as important anti-cancer effect [82, 83] that promotes cell death. However, the debate surrounding the metabolic dependency of PDAC and PCa remains unresolved, and a better understanding of metabolic features is crucial to identify more effective therapeutic approaches to prolong overall and progression-free survival of patients. The results of this study provide support at molecular level for the use of FASN inhibition in PCa in future treatment strategies.

## Conclusions

In this study, we used bioinformatics and cell-based models to systematically analyze the expression, mutations, and prognostic and metabolic role of FASN in PDAC and PCa. We found that FASN expression differs in PDAC and PCa. The higher FASN expression levels observed in PCa, associated with lower methylation at its promoter, seem to affect the aggressiveness of the disease, while the greater sensitivity of PCa to FASN inhibition may provide new therapeutic insights.

## Supplementary Information

**Additional file 1: Figure S1.** Fatty acid synthase (FASN) transcription levels in cancer (red) and normal tissue (blue). mRNA expression in various cancer types was obtained from the GENT database. Y-axis shows log2 fold change values, while X axis shows tissue types. Boxes indicate the median, and 25th and 75th percentiles. Red boxes indicate tumor tissues; blue boxes indicate normal tissues. **Figure S2.** A Gene query showing clustered FASN expression in tumors expressed as TPM. B Bulk tissue gene expression for FASN. Data is shown as a violin plot and on a log scale, sorted by expression, while dots represent outliers. Y-axis shows log2 fold change values, while X-axis shows tissue types. Data were obtained from the GTEx portal database (https://gtexportal.org). **Figure S3.** RNA expression of FASN in cancer. Data is shown as a box plot with FPKM values, while dots represent outliers. Data were obtained from The Human Protein Atlas using pathological classification. **Figure S4.** A Gene query showing clustered FASN expression of all transcripts in tumors expressed as TPM. B-C show accessibility of exons (red labels) and expression of each transcript (blue labels) in PDAC and PCa, respectively. Data were obtained from the GTEx portal database. **Figure S5.** A-B Overall survival prognosis based on FASN expression (high = red, low = blue) in PDAC and PCa. Data were obtained from UALCAN and cBioPortal platforms, respectively. **Figure S6.** Alteration frequ ency of FASN gene from the cBioPortal database indicated as mutations (green), fusions (purple), amplifications (red), deep deletions (blue), and multiple alterations (gray). **Figure S7.** A-B Scatter plots of FASN expression (X-axis) and SREBF1 (Y-axis) in PDAC and PCa. C-D Heatmaps showing Pearson correlation related to co-expression of FASN-SREBF1. Data were downloaded from UALCAN. **Figure S8.** Graphs showing IC50 for TVB-2640 in PL-45, SW1990, LNCaP, and C4-2 cells at 24 h, 48 h, and 72 h. **Figure S9.** Oil-Red O staining results of the lipid drop accumulation assay in LNCaP in control and after treatment with TVB-2640 50 μM. **Figure S10.** Colony assay in LNCaP cells, A) control and B) treated with TVB-2640 50 μM for 72 h.

**Additional file 2.** FASN Gene Expression across Normal and Tumor tissue in different human cancers. Data provide from GPL96 and GPL570 microarray databases and reported as expression level.

**Additional file 3.** FASN Gene Expression in PCa and PDAC patients. Data provide from GPL96 and GPL570 microarray databases and reported as expression level.

**Additional file 4.** Mutation profiles for FASN in PCa and PDAC patients. Data, such as variant type, mutation type, amino acid change, provide from TCGA database.

**Additional file 5.** Gene expression correlation between FASN and SREBF1 in PCa and PDAC patients. Data provide from TCGA database reported as expression level.

**Additional file 6.** Functional association data documenting both physical and functional protein–protein interactions for FASN and FASN-related interactors. Data provide from STRING database and listed based on score.

## Data Availability

Data are available on request from the authors. The data that support the findings of this study are available from the corresponding author, R.B., upon reasonable request.

## Abbreviations

FA: Fatty acid
FASN: FA synthase
GSEA: Gene set enrichment analysis
NT: Normal tissue
PDAC: Pancreatic ductal adenocarcinoma
PCa: Prostate cancer
