# Supplementary material for: FASN multi-omic characterization reveals metabolic heterogeneity in pancreatic and prostate adenocarcinoma
Source: J Transl Med. 2023 Jan 17;21:32. doi: 10.1186/s12967-023-03874-5 (PMC9847120; doi:10.1186/s12967-023-03874-5)
Supplement: Supplementary file 1 — Additional file 1: Figure S1. Fatty acid synthase (FASN) transcription levels in cancer (red) and normal tissue (blue). mRNA expression in various cancer types was obtained from the GENT database. Y-axis shows log2 fold change values, while X axis shows tissue types. Boxes indicate the median, and 25th and 75th percentiles. Red boxes indicate tumor tissues; blue boxes indicate normal tissues. Figure S2. A Gene query showing clustered FASN expression in tumors expressed as TPM. B Bulk tissue gene expression for FASN. Data is shown as a violin plot and on a log scale, sorted by expression, while dots represent outliers. Y-axis shows log2 fold change values, while X-axis shows tissue types. Data were obtained from the GTEx portal database (https://gtexportal.org). Figure S3. RNA expression of FASN in cancer. Data is shown as a box plot with FPKM values, while dots represent outliers. Data were obtained from The Human Protein Atlas using pathological classification. Figure S4. A Gene query showing clustered FASN expression of all transcripts in tumors expressed as TPM. B-C show accessibility of exons (red labels) and expression of each transcript (blue labels) in PDAC and PCa, respectively. Data were obtained from the GTEx portal database. Figure S5. A-B Overall survival prognosis based on FASN expression (high = red, low = blue) in PDAC and PCa. Data were obtained from UALCAN and cBioPortal platforms, respectively. Figure S6. Alteration frequ ency of FASN gene from the cBioPortal database indicated as mutations (green), fusions (purple), amplifications (red), deep deletions (blue), and multiple alterations (gray). Figure S7. A-B Scatter plots of FASN expression (X-axis) and SREBF1 (Y-axis) in PDAC and PCa. C-D Heatmaps showing Pearson correlation related to co-expression of FASN-SREBF1. Data were downloaded from UALCAN. Figure S8. Graphs showing IC50 for TVB-2640 in PL-45, SW1990, LNCaP, and C4-2 cells at 24 h, 48 h, and 72 h. Figure S9. Oil-Red O staining results of t [file 12967_2023_3874_MOESM1_ESM.pdf]

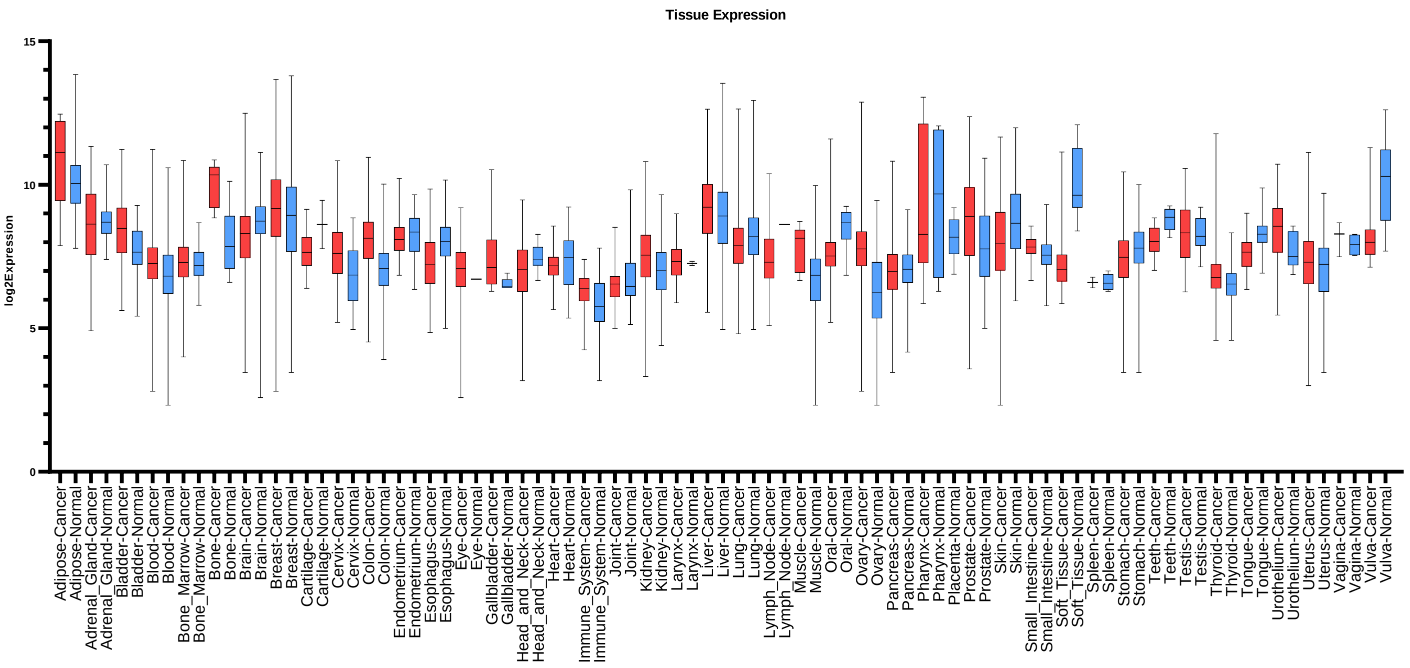

Supplementary Figure 1: Fatty acid synthase (FASN) transcription levels in cancer (red) and normal tissue (blue). mRNA expression in various cancer types was obtained from the GENT database. Y-axis shows log2 fold change values, while X-axis shows tissue types. Boxes indicate the median, and 25th and 75th percentiles. Red boxes indicate tumor tissues; blue boxes indicate normal tissues.

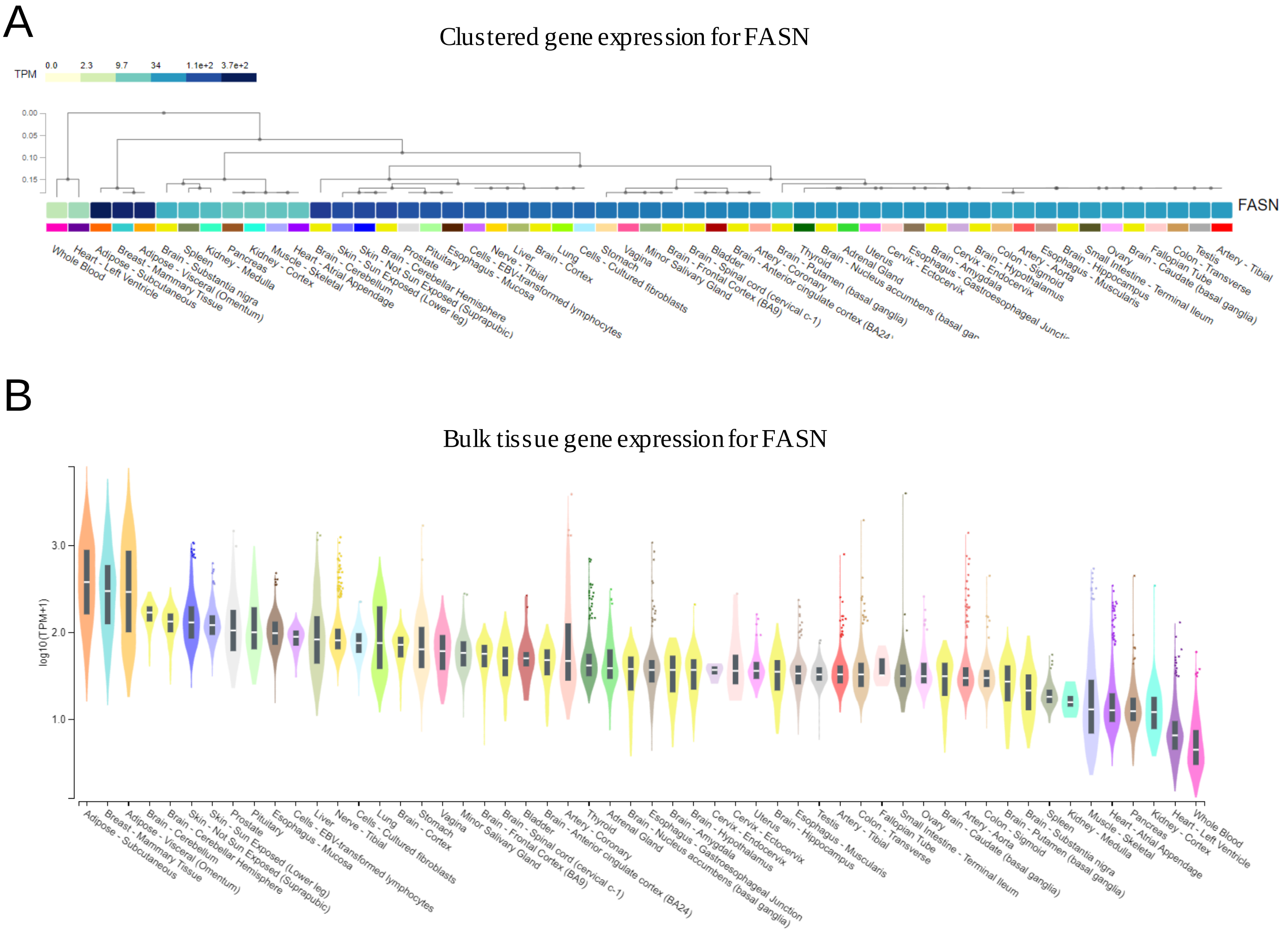

Supplementary Figure 2: **A** Gene query showing clustered FASN expression in tumors expressed as TPM. **B** Bulk tissue gene expression for FASN. Data is shown as a violin plot and on a log scale, sorted by expression, while dots represent outliers. Y-axis shows log2 fold change values, while X-axis shows tissue types. Data were obtained from the GTEx portal database (https://gtexportal.org).

TCGA dataset: RNA cancer

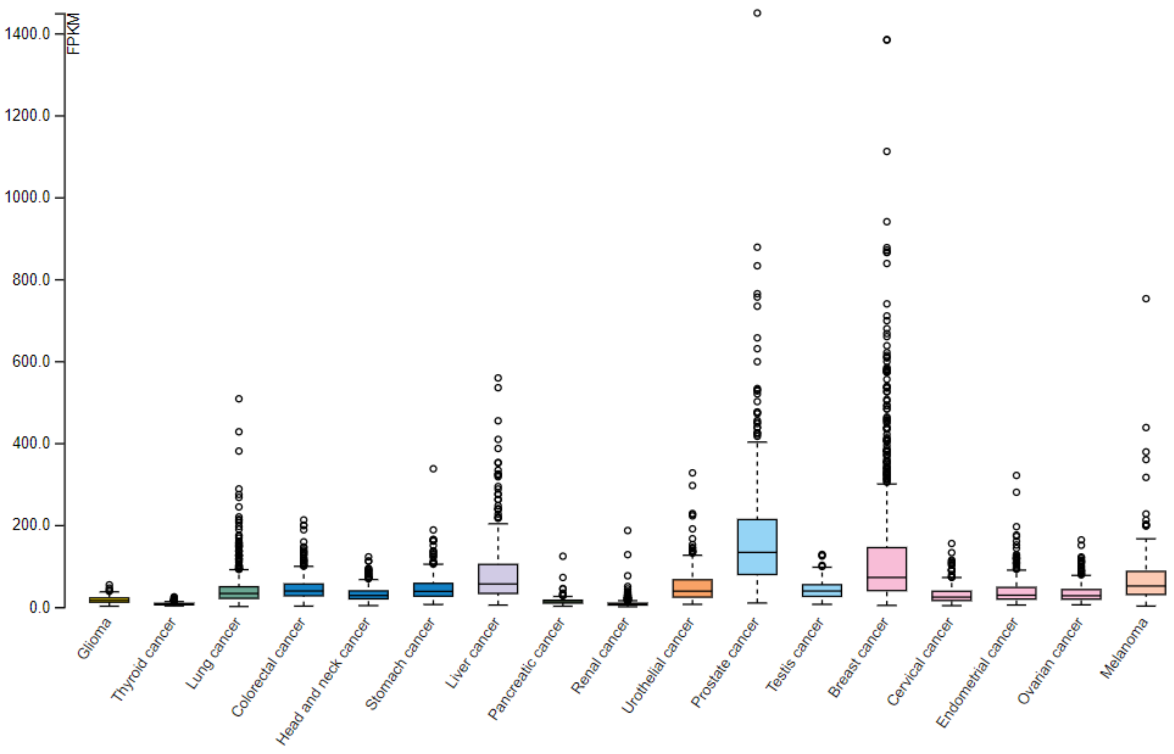

Supplementary Figure 3: RNA expression of FASN in cancer. Data is shown as a box plot with FPKM values, while dots represent outliers. Data were obtained from The Human Protein Atlas using pathological classification.

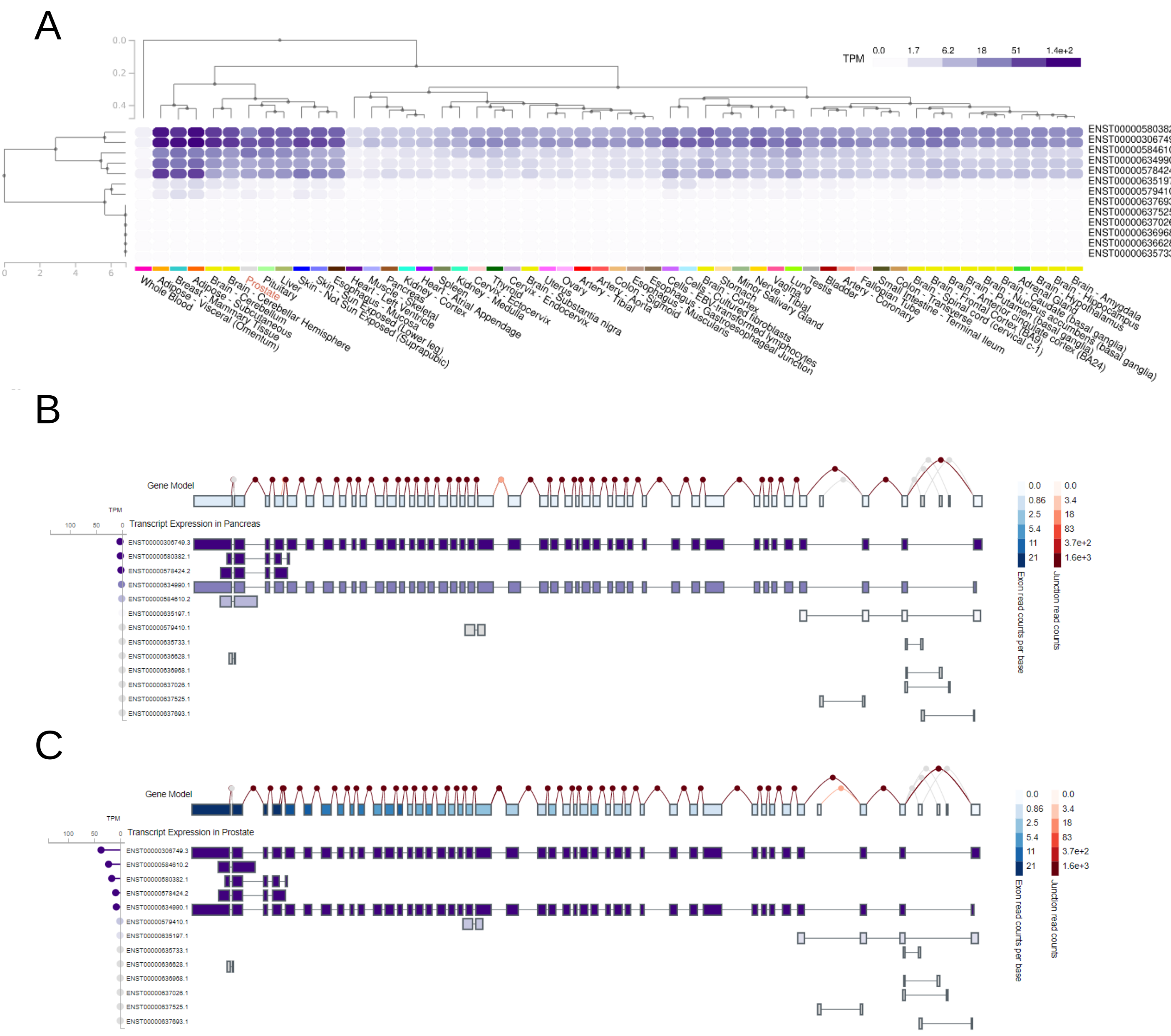

Supplementary Figure 4: A Gene query showing clustered FASN expression of all transcripts in tumors expressed as TPM. **B-C** show accessibility of exons (red labels) and expression of each transcript (blue labels) in PDAC and PCa, respectively. Data were obtained from the GTEx portal database. .

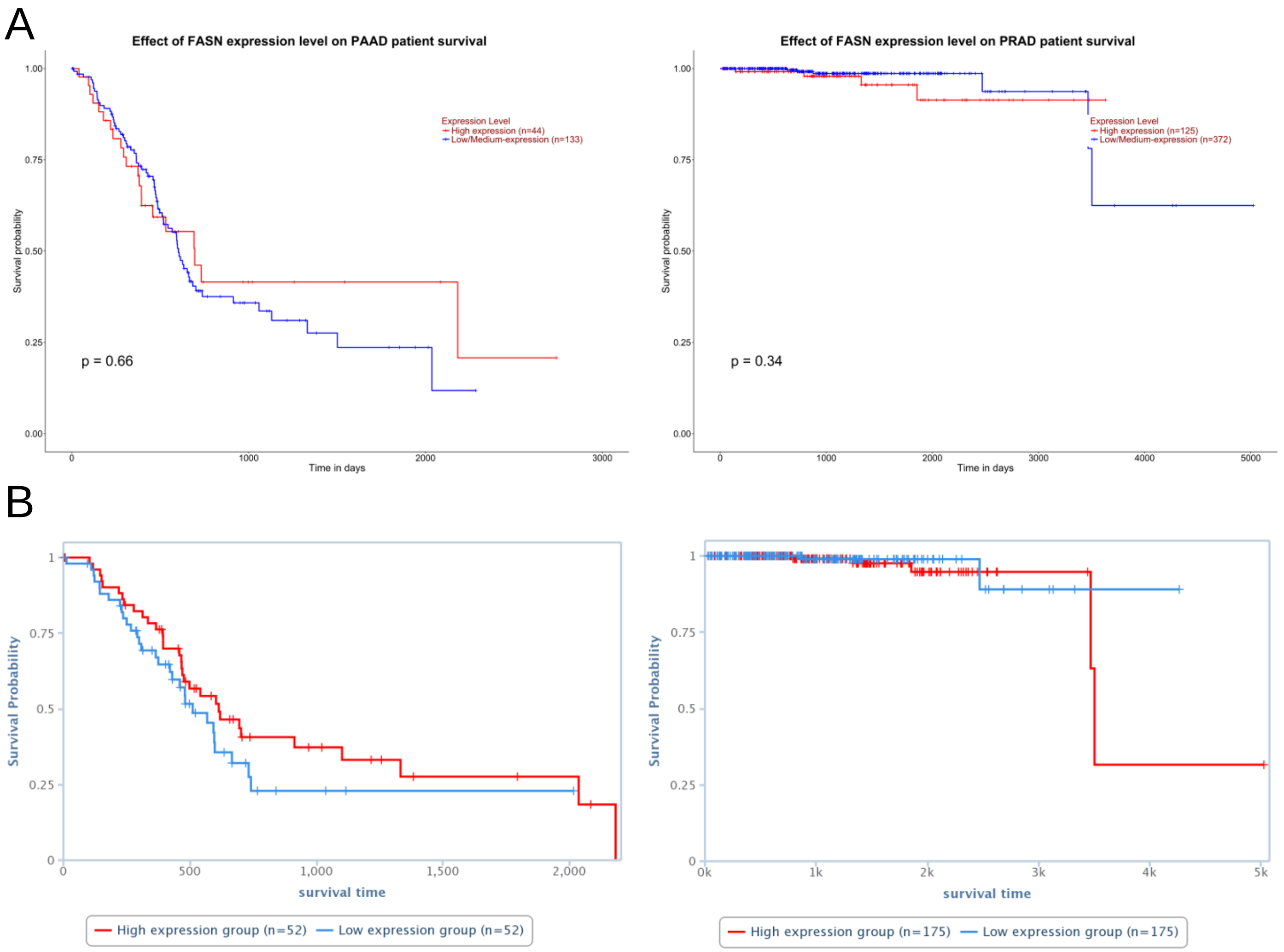

Supplementary Figure 5: **A-B** Overall survival prognosis based on FASN expression (high=red, low=blue) in PDAC and PCa. Data were obtained from UALCAN and cBioPortal platforms, respectively.

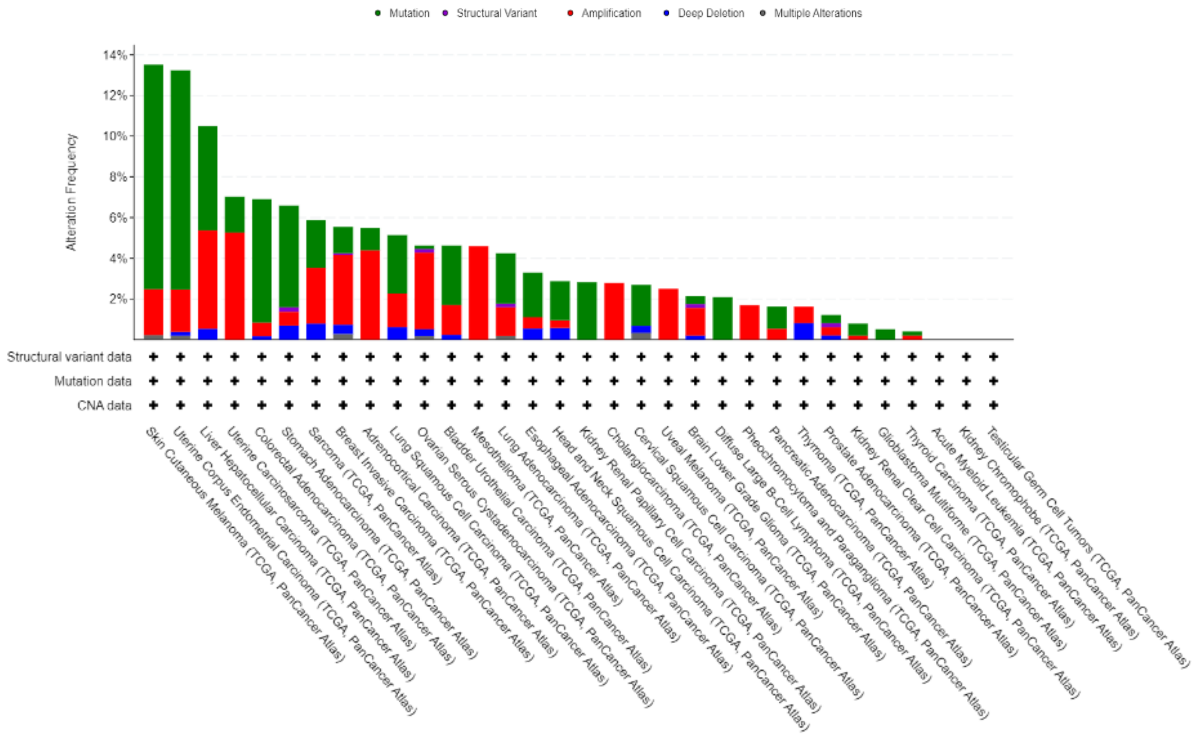

Supplementary Figure 6: Alteration frequency of FASN gene from the cBioPortal database indicated as mutations (green), fusions (purple), amplifications (red), deep deletions (blue), and multiple alterations (gray).

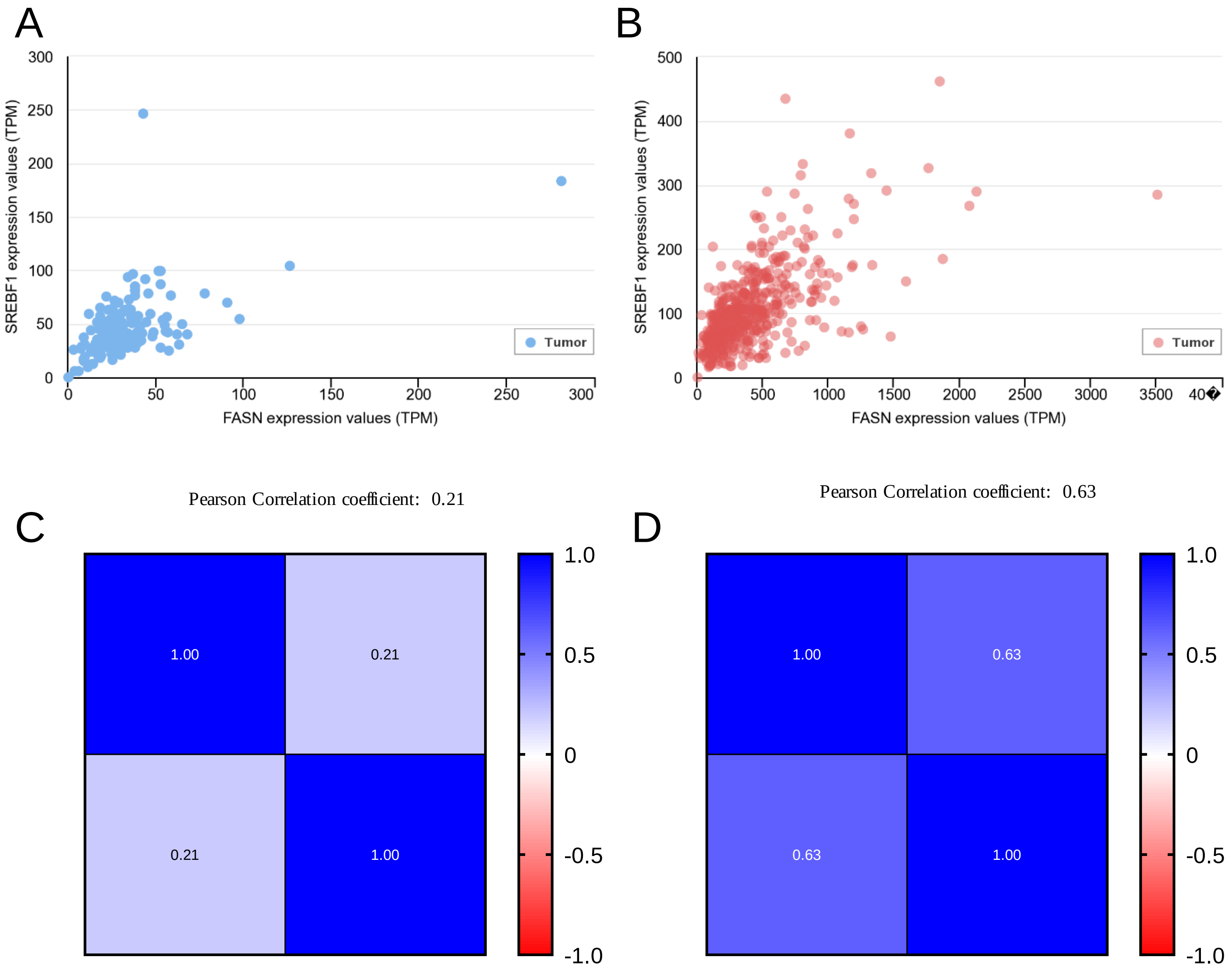

Supplementary Figure 7: A-B Scatter plots of FASN expression (X-axis) and SREBF1 (Y-axis) in PDAC and PCa. **C-D** Heatmaps showing Pearson correlation related to co-expression of FASN-SREBF1. Data were downloaded from UALCAN..

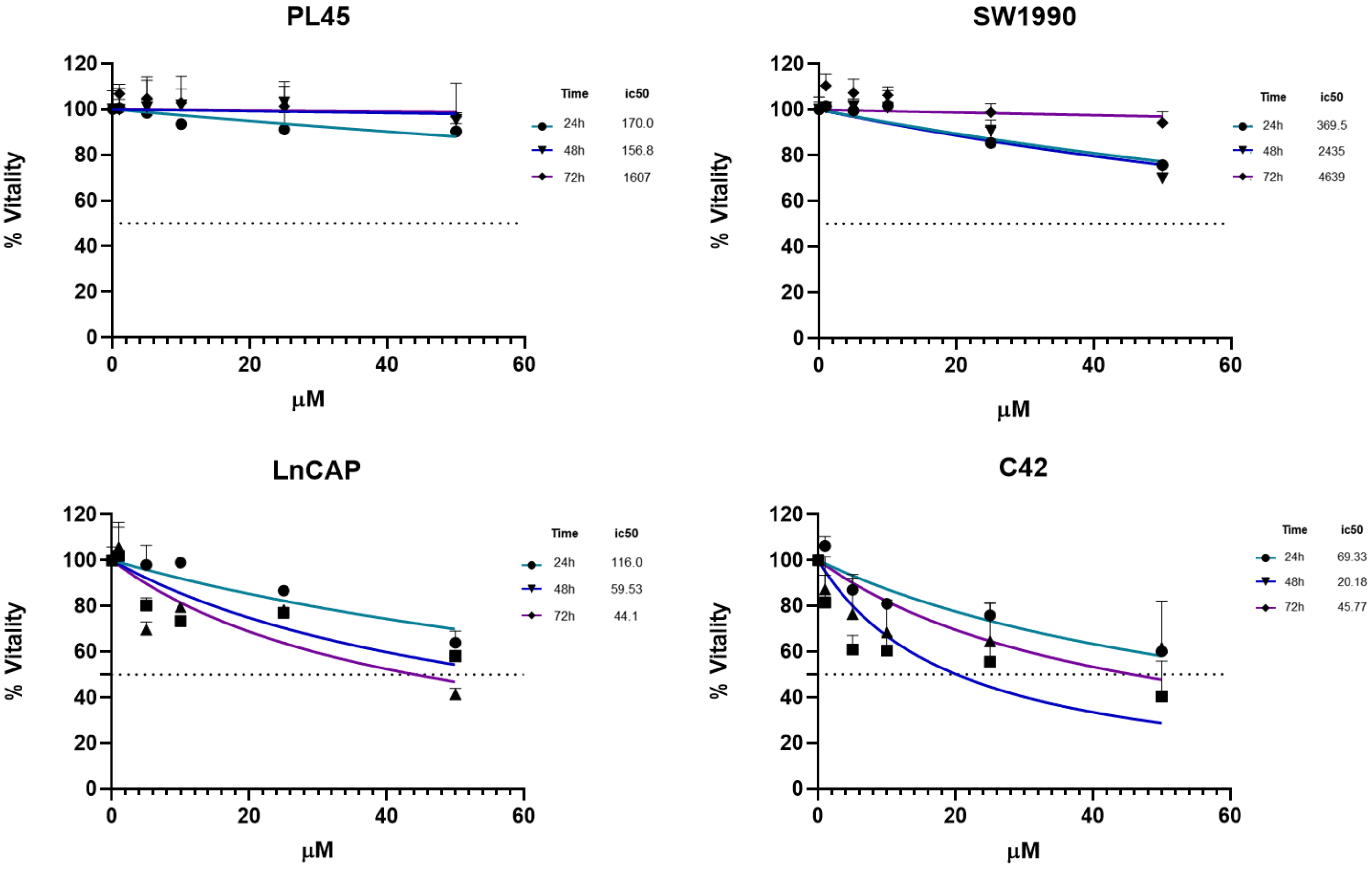

Supplementary Figure 8: Graphs showing IC50 for TVB-2640 in PL-45, SW1990, LNCaP, and C4-2 cells at 24 h, 48 h, and 72 h.

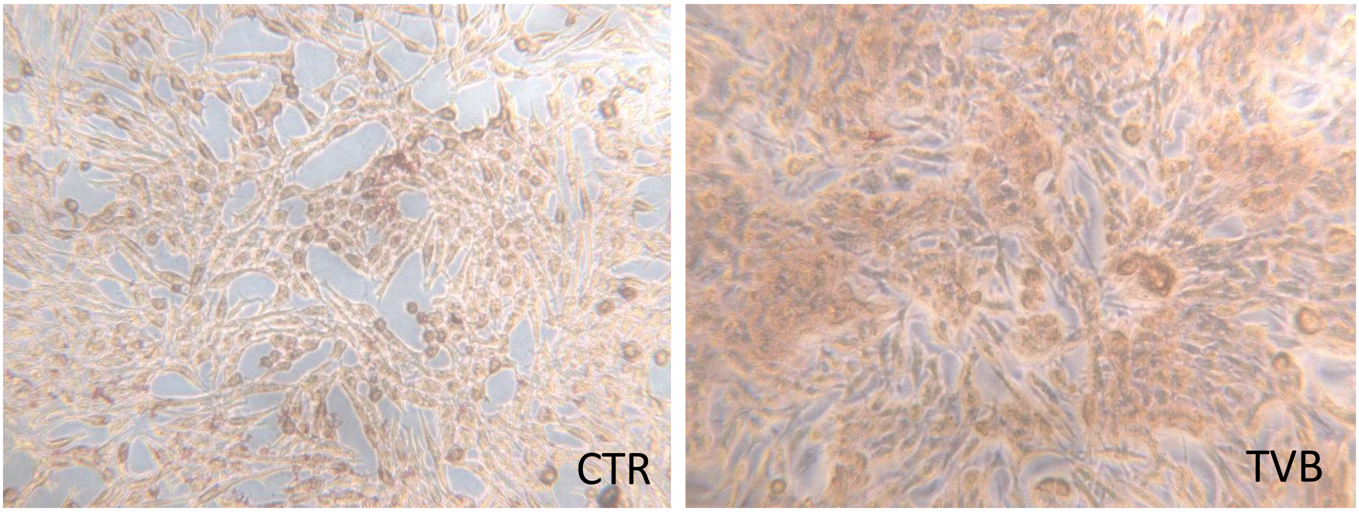

Supplementary Figure 9: Oil-Red O staining results of the lipid drop accumulation assay in LNCaP in control and after treatment with TVB-2640 50 μM for 24h. Image acquisition has been done with light microscope 20X magnification.

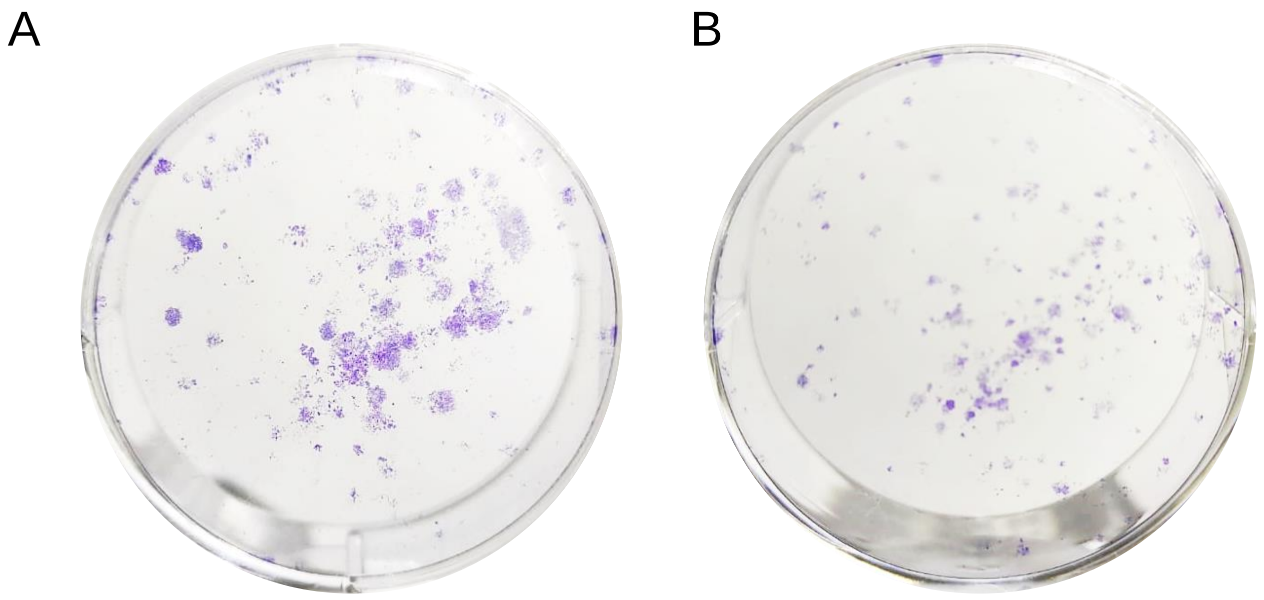

Supplementary Figure 10: Colony assay in LNCaP cells, **A**) control and **B**) treated with TVB-2640 50 μM for 72 h.
